# SARS-CoV-2-mRNA Booster Vaccination Reverses Non-Responsiveness and Early Antibody Waning in Immunocompromised Patients – A Phase Four Study Comparing Immune Responses in Patients With Solid Cancers, Multiple Myeloma and Inflammatory Bowel Disease

**DOI:** 10.3389/fimmu.2022.889138

**Published:** 2022-05-12

**Authors:** Angelika Wagner, Erika Garner-Spitzer, Anna-Margarita Schötta, Maria Orola, Andrea Wessely, Ines Zwazl, Anna Ohradanova-Repic, Lukas Weseslindtner, Gabor Tajti, Laura Gebetsberger, Bernhard Kratzer, Elena Tomosel, Maximilian Kutschera, Selma Tobudic, Winfried F. Pickl, Michael Kundi, Hannes Stockinger, Gottfried Novacek, Walter Reinisch, Christoph Zielinski, Ursula Wiedermann

**Affiliations:** ^1^ Institute of Specific Prophylaxis and Tropical Medicine, Center for Pathophysiology, Infectiology and Immunology, Medical University Vienna, Vienna, Austria; ^2^ Institute for Hygiene and Applied Immunology, Center for Pathophysiology, Infectiology and Immunology, Medical University Vienna, Vienna, Austria; ^3^ Center for Virology, Medical University of Vienna, Vienna, Austria; ^4^ Institute of Immunology, Center for Pathophysiology, Infectiology and Immunology, Medical University Vienna, Vienna, Austria; ^5^ Department of Medicine III, Division of Gastroenterology and Hepatology, Medical University Vienna, Vienna, Austria; ^6^ Department of Medicine I, Division of Infectious Diseases and Tropical Medicine, Medical University of Vienna, Vienna, Austria; ^7^ Center for Public Health, Medical University Vienna, Vienna, Austria; ^8^ Central European Cancer Center, Wiener Privatklinik, Vienna, Austria; ^9^ The Central European Cancer Center, Central European Cooperative Oncology Group, Headquater (HQ), Vienna, Austria

**Keywords:** patients under immunosuppression/immunomodulation, SARS-CoV-2 mRNA vaccination, booster vaccination, humoral and cellular vaccine-specific responses, antibody testing, waning of immune responses, anamnestic vaccine-specific response

## Abstract

**Background:**

Individuals with secondary immunodeficiencies belong to the most vulnerable groups to succumb to COVID-19 and thus are prioritized for SARS-CoV-2 vaccination. However, knowledge about the persistence and anamnestic responses following SARS-CoV-2-mRNA vaccinations is limited in these patients.

**Methods:**

In a prospective, open-label, phase four trial we analyzed S1-specific IgG, neutralizing antibodies and cytokine responses in previously non-infected patients with cancer or autoimmune disease during primary mRNA vaccination and up to one month after booster.

**Results:**

263 patients with solid tumors (SOT, n=63), multiple myeloma (MM, n=70), inflammatory bowel diseases (IBD, n=130) and 66 controls were analyzed. One month after the two-dose primary vaccination the highest non-responder rate was associated with lower CD19^+^ B-cell counts and was found in MM patients (17%). S1-specific IgG levels correlated with IL-2 and IFN-γ responses in controls and IBD patients, but not in cancer patients. Six months after the second dose, 18% of patients with MM, 10% with SOT and 4% with IBD became seronegative; no one from the control group became negative. However, in IBD patients treated with TNF-α inhibitors, antibody levels declined more rapidly than in controls. Overall, vaccination with mRNA-1273 led to higher antibody levels than with BNT162b2. Importantly, booster vaccination increased antibody levels >8-fold in seroresponders and induced anamnestic responses even in those with undetectable pre-booster antibody levels. Nevertheless, in IBD patients with TNF-α inhibitors even after booster vaccination, antibody levels were lower than in untreated IBD patients and controls.

**Conclusion:**

Immunomonitoring of vaccine-specific antibody and cellular responses seems advisable to identify vaccination failures and consequently establishing personalized vaccination schedules, including shorter booster intervals, and helps to improve vaccine effectiveness in all patients with secondary immunodeficiencies.

**Trial registration:**

EudraCT Number: 2021-000291-11

## Introduction

Infections with the SARS-CoV-2 virus may lead to severe disease in the immunosuppressed population (1, 2). COVID-19 vaccines have been developed to combat the ongoing pandemic by preventing particularly severe courses of disease. These vaccines have been shown to be highly immunogenic in healthy individuals (3, 4). However, immunogenicity and vaccine efficacy in patients under different immunosuppressive/-modulatory treatments are still a matter of concern. Humoral and/or cellular immune responses are generally affected to different degrees depending on the underlying disease and treatment regimens. Thus, impaired vaccine responses were described for cancer patients in particular under chemotherapy and with progressive disease as well as with low lymphocyte counts around four weeks after the second vaccine dose (5–7). With regard to inflammatory bowel disease (IBD), it has been shown that the type of treatment - particularly with TNF-α-inhibitors - results in decreased immunogenicity, when measured up to 2 weeks after the second vaccine dose, as compared to healthy individuals, although shown in an only small cohort (8).

Magnitude of response may also be related to the type of mRNA vaccine used, as for patients with anti-CD20 therapy or with multiple myeloma better seroconversion rates have been reported following the administration of mRNA-1273 (Moderna) rather than of BNT162b2 (Pfizer BioNTech) (6, 9). Very little data is available regarding the vaccine-specific cellular responses and whether a correlation or dissociation of humoral and cellular immune response can predict vaccine responsiveness/non-responsiveness and subsequent protection or failure to protect against disease. Another unanswered question is the persistence of immune responses in seropositive immunosuppressed patients upon two doses of mRNA vaccines and response to booster vaccines.

Here, we present data obtained from patients with cancers (solid tumor (SOT) and multiple myeloma (MM)) as well as with IBD receiving different immunosuppressive/-modulatory treatments compared to healthy controls without previous SARS-CoV-2 infection. We characterized the correlation between antibody and cellular responses and investigated if vaccine (non)-responsiveness can be predicted by the leukocyte distributions prior to primary vaccination. Furthermore, we evaluated the persistence and waning of antibody levels in immunocompromised patients up to six months after primary vaccination and to which extent vaccine failures can be reverted by booster doses. Finally, we differentiated between BNT162b2- and mRNA-1273-vaccinated individuals to assess if favorable use of one vaccine over the other might be considered.

## Material and Methods

### Study Population

We invited patients without prior COVID-19 vaccination suffering from solid tumors (SOT), multiple myeloma (MM), inflammatory bowel disease (IBD) as well as healthy individuals (controls) *via* the outpatient vaccination clinic at the Institute of Specific Prophylaxis and Tropical Medicine and the Department of Internal Medicine of the Medical University Vienna and General Hospital of Vienna to participate in this study. Recruitment started in March 2021 and we enrolled a volunteer sample. In total, 329 participants were enrolled and 263 with various immunosuppressive/-modulatory treatments were included for the final analysis, excluding those participants with prior COVID-19 infection, with incomplete two-dose mRNA vaccination schedule or lost to follow-up (**Supplementary Figures 1, 2**). All 329 participants had received two doses of a mRNA vaccine (either BNT162b2, Comirnaty, Pfizer BioNTech or mRNA-1273, Spikevax, Moderna Biotech) *via* the Austrian COVID-19 Immunization campaign where two doses were administered in intervals of three and four weeks, respectively. Serum samples for antibody titers measurements were collected before the first dose, on the day of the second dose and four weeks as well as five to six months after the second dose. After participants had received their booster dose they were invited for a blood draw four weeks later. Sera were frozen and stored until analysis. Participants without antibody responses four weeks after the second dose (SOT: n=1; MM: n=12) were offered an earlier third dose and therefore were not further included in the analysis of antibody responses five to six months after the second dose. In a subgroup (n=102) and upon participants consent lithium heparinized blood was taken before the first dose and seven days after the second dose to isolate peripheral blood mononuclear cells (PBMC). The study was conducted as an investigator-initiated prospective, open-label, phase four trial involving three parallel patient groups and a control group during primary vaccination and one month after booster.

We obtained written informed consent from all participants before inclusion according to the Declaration of Helsinki/International Conference on Harmonisation Guideline for Good Clinical Practice. The study was registered with the European Union Clinical Trial Register and approved by the Ethics Committee of the Medical University of Vienna (EK: 1073/2021).

### Study Procedures

Blood samples were drawn before the first and the second dose, four weeks and five-six months after the second dose (i.e. before the third dose) as well as four weeks after the third dose and serum samples used for antibody measurements. PBMCs were obtained from blood samples before and seven days after primary vaccination to analyze T cell responses. Time points to measure antibody levels four weeks after primary vaccination and T cell responses seven days after primary vaccination were chosen based on previous studies on humoral and cellular responses to different vaccine antigens (10–12).

### Humoral Responses

SARS-CoV-2-specific IgG antibodies directed against the subunit 1 (S1) of the spike protein were measured by ELISA (Quantivac^®^, Euroimmun) in diluted serum samples (1:101) according to the manufacturer´s instructions. Antibody quantification results are expressed in binding antibody units/ml (BAU/ml) with values above 35.2 BAU/ml considered positive as defined by the manufacturer.

Neutralization assay was performed in the preselected subgroup for cellular evaluation with the human SARS-CoV-2 isolate BetaCoV/Munich/BavPat1/2020 kindly provided by Prof. Christian Drosten, Charité, Berlin, and distributed by the European Virology Archive (Ref-SKU: 026V-03883) (13). The virus was passaged once through the human lung adenocarcinoma cell line Calu-3 (ATCC HTB-55TM, kindly provided by Prof. Walter Berger, Medical University of Vienna, Vienna, Austria) to obtain a high titer virus stock. Neutralization assay was performed according to the protocol by Amanat et al. (14). Briefly, one day prior the assay, 10000 Vero cells (ATCC CCL-81TM, kindly provided by Prof. Sylvia Knapp, Medical University of Vienna, Vienna, Austria) were seeded into each well of a 96-well plate (in a Dulbecco´s Modified Eagle´s medium, DMEM, Gibco/Thermo Fisher, high glucose, with GlutaMAX and sodium pyruvate, supplemented with 10% fetal calf serum, FCS, Biowest, Nuaillé, France; 1% MEM non-essential amino acids solution, Gibco/Thermo Fisher; 100 U/ml penicillin and 100 μg/ml streptomycin, Gibco/Thermo Fisher). On the next day, paired pre-immune and immune heat-inactivated (56°C for 30 min) proband sera were serially diluted in DMEM medium with reduced serum (2% FCS) and incubated in duplicates with the SARS-CoV-2 virus (80 μl, equaling 800 half-maximal tissue culture infectious dose (TCID50) per well; final volume 160 µl per well) for 1 hour at 37°C in a Biosafety Level 3 facility of the Medical University of Vienna. After one hour, 120 µl of the mixture was used to infect the monolayers of Vero cells, achieving infection of 600 TCID50/well. 48 hours later, infected cells were fixed with 10% formaldehyde in PBS, followed by 5% formaldehyde postfix. Subsequently, cells were washed with PBS, permeabilized with 0.1% Triton X-100 detergent in PBS, blocked with a blocking buffer (10% FCS in PBS+0.05% Tween-20 detergent), and stained with a rabbit anti-SARS-CoV-2 nucleocapsid monoclonal antibody (40143-R019, SinoBiological, Beijing, China) diluted 1:15000 in blocking buffer), followed by the goat anti-rabbit-HRP antibody (170-6515, Bio-Rad, diluted 1:10000 in blocking buffer). In-Cell ELISA was then developed using DY999 substrate solution according to the manufacturer’s recommendations (R&D Systems, Minneapolis, MN, USA) and measured at 450 nm (and at 630 nm to assess the background) using a Mithras multimode plate reader (Berthold Technologies, Bad Wildbad, Germany). To calculate percent neutralization at each well, the following formula was used: 100 − [(X - average of ‘no virus’ wells)/(average of ‘virus only’ wells - average of ‘no virus’ wells)*100], where X is the background-subtracted read for each well. Titers were calculated in GraphPad Prism 9 by generating a 4-parameter logistic fit of the percent neutralization at each serial serum dilution. The 50% virus neutralization titer (NT50) was reported as the interpolated reciprocal of the dilution yielding a 50% reduction in the anti-SARS-CoV-2 nucleocapsid staining.

Surrogate neutralization assays (sVNT, cPass, GenScript), recognizing the original D614G virus strain, were performed in a randomly selected subset of 20 participants per group before and after booster vaccination according to the manufacturer´s instructions using a cut-off for positivity at 30% RBD/ACE binding inhibition. High levels of neutralizing antibodies with inhibition greater than 93.8% correlated with a 99% probability of neutralizing antibody titers ≥1:80 by a live virus neutralization assay using a protocol previously described (15).

### Leukocyte Phenotyping

Phenotyping of leukocyte subpopulations in whole blood samples was performed by flow cytometric analysis after staining with directly-conjugated monoclonal antibodies, as previously described (16) calculating absolute cell counts.

### T-Cell Responses

PBMCs were isolated from heparinized human peripheral blood *via* density gradient centrifugation using Ficoll™ (LSM 1077, PAA, Pasching, Austria) as previously described (17). For each participant, we seeded 190 µl medium containing 5 x 10^5^ of the freshly isolated PBMCs into each of four wells of a 96-well plate (Cellstar Cat.-No.: 650 180, Greiner Bio-One GmbH, Kremsmünster, Austria). The cultivation medium used was RPMI 1640 supplemented with 2 mM L-glutamine (LonzaTM, BioWhittakerTM, Fisher Scientific, Vienna, Austria) and 5% human AB serum (PAN-Biotech Seraclot, Aidenbach, Germany). The cells were rested over night before adding the stimulants. Two different SARS-CoV-2 specific stimulants were used, namely the PepTivator^®^ SARS-CoV-2 peptide pools of the S1 domain of the spike protein and of the nucleocapsid protein N (Cat. No. 130-127-041 and 130-126-698) Miltenyi Biotech, Bergisch Gladbach, Germany). The lyophilized antigens were reconstituted in sterile water (Cat. No. 95284, Merck, Darmstadt, Germany) as described in the manufacturer’s instructions yielding a stock concentration of 30 nmol of each peptide per ml. Working aliquots with a concentration of 3 nmol of each peptide per ml in 1 x DPBS (GibcoTM Cat. No. 14190094, Thermo Fisher Scientific, Vienna, Austria) were prepared and stored at -80°C until usage. Rested PBMC were stimulated with 10 µl of these working solutions resulting in a stimulation with 0.03 nmol per peptide per 5 x 10^5^ cells in a final volume of 200 µl. In parallel, stimulation with 10 µl of 1 x DPBS or with 10 µl of PHA (100 µg/ml working solution; 5 µg/ml final concentration) was performed as a negative or positive control, respectively. The cells were stimulated for 24 hours after which the supernatant was taken and stored at -80°C until cytokine measurement. For the latter, we used the Luminex^®^ 100/200 System and determined the concentration of the following cytokines: interleukin (IL)-2, IL-5, IL-10, IL-17a, IL-22, granulocyte-macrophage colony-stimulating factor (GM-CSF) and interferon (IFN)-γ using Luminex Human High-Sensitive Cytokine Performance Assays from Bio-Techne (Minneapolis, MN, USA) according to the manufacturer’s instructions.

### Statistical Analysis

Sample size was determined by estimating seroconversion rates from influenza vaccination in immunocompromised versus healthy individuals with two sided significance level of 0.05 and a power of 0.8.

The primary outcome was defined as the seroconversion achieved four weeks after two COVID-19 mRNA doses in the different patient groups and the control group based on SARS-CoV-2-specific antibody measurements. Secondary outcomes included the antibody levels measured before the first dose and five-six months after the second as well as four weeks after a booster dose, cellular SARS-CoV-2-specific immune responses before the first dose and one week after the second dose measured by T cell cytokine production of restimulated PBMC and differences in humoral and cellular responses between the groups.

Seroprevalence data are presented as descriptive statistics. Average antibody levels are expressed as geometric mean concentrations (GMC) or geometric mean titers (GMT). To evaluate differences between the different study groups, we compared results with non-parametric Kruskal-Wallis test including Dunn´s multiple comparisons test. Relationships between humoral and cellular responses were assessed by linear regression analysis. Comparison of cytokine production before the first and after the second dose and decrease of antibody levels between four weeks and five-six months after the second vaccine dose were calculated by paired t-test after log transformation. P-values <0.05 were considered significant.

## Results

### Demographic Data

Between March 2021 und June 2021, 263 patients (55.9% females, mean age 54.5 ± 16.2) with underlying SOT (n=63; 83.3% females, mean age 62.8 ± 11.5) of the breast (71%) or lung (29%), MM (n=70; 44.3% females, mean age 66.5 ± 8.0) or IBD (n=130; 50.0% females, mean age 46.1 ± 15.1) undergoing different treatment regimens and 66 controls (50% females, mean age 46.1 ± 15.1) were included (**Table 1**, **2**).

**Table 1:** Description of the study population (n=263 patients, n=66 controls).

	SOT (n=63)	MM (n=70)	IBD (n=130)	controls (n=66)
Age (y) mean ± SD; (range)	62.8 ± 11.5; (38–82)	66.5 ± 8.0; (46-83)	44.0 ± 14.4; (19-77)	46.1 ± 15.1; (20-78)
mean BMI ± SD	24.8 ± 5.0	26.1 ± 4.0	31.4 ± 6.7	25.5 ± 4.5
obese (BMI > 30) n (%)	7 (11.0)	12 (17.1)	12 (9.2)	12 (18.2)
Female n (%)	55 (87.3)	31 (44.3)	61 (46.9)	33 (50.0)
Mean time from diagnosis (y)	5.3 ± 7.1	8.3 ± 6.9	15.0 ± 10.7	n.a.
Vaccine				
BNT162b2 n (%)	36 (57.1)	48 (68.6)	2 (1.5)	5 (7.6)
mRNA-1273 n (%)	27 (42.9)	22 (31.4)	128 (98.5)	61 (92.4)
Ongoing immunosuppressive/- immunomodulator treatment n (%)	49 (77.8)	45 (64.3)	111 (83.8)	n.a.
Stem cell transplantation n (%)	n.a.	32 (45.7)	n.a.	n.a.

n.a. (not applicable).

**Table 2:** Characteristics of IBD patients.

IBD type	n (%)	Gender f: n (%)	Age mean ± SD (range)	Time from diagnosis in years ± SD	Ongoing immuno-suppressive/-modulatory treatment n (%)
Total	130	61 (46.9)	19-77	15.0 ± 10.7	111 (83.8)
Crohn´s disease (CD)	81 (62.3)	36 (44.4)	44.0 ± 12.8 (19-77)	16.4 ± 10.5	70 (86.4)
Ulcerative colitis (UC)	48 (36.9)	25 (52.1)	44.6 ± 16.6 (20-74)	13.3 ± 10.7	39 (81.3)
IBD unclassified (IBDU)	1 (0.8)	0 (0)	23.0 ± 0.0 (23)	3.0 ± 0.0	0 (0)

### Humoral Immune Responses After Primary Vaccination

**Figure 1:**
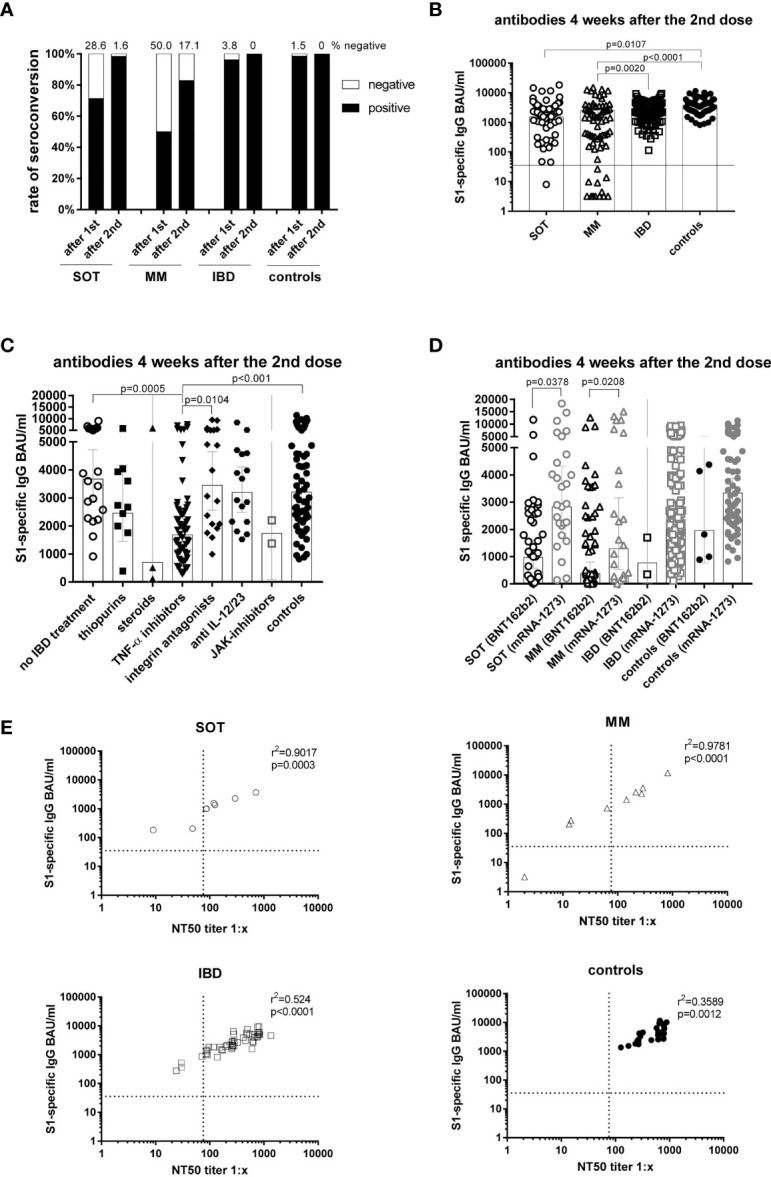
Antibody responses four weeks after the second mRNA vaccination and correlation of S1-specific IgG with neutralizing antibodies. Seroconversion rates after the first and second dose in all study participants of all groups **(A)**. Individual S1-specific IgG levels of all participants **(B)**. S1-specific IgG levels of IBD patients in respect of their treatment and in comparison to the controls **(C)**. S1-specific antibody levels in relation to the type of mRNA vaccine applied (BNT162b2 or mRNA-1273), whereby due to the number of participants statistical differences could only be calculated for SOT and MM patients **(D)**. Correlation of S1-specific IgG levels with the NT50 of a neutralization test with sera taken four weeks after the second vaccination (n=106) **(E)**. SOT (n=63) are represented as circles, MM (n=70) as triangles, IBD (n=130) patients as squares and controls (n=55) as full black or grey circles. Differences between the groups below p values of 0.05 were regarded as significant. The black and dotted lines **(E)** indicate the threshold for positive results (35.2 BAU/ml and NT50). Bars **(B-D)** represent GMC with 95% confidence interval (CI).

After the first dose, 50.0% of MM, 28.6% of SOT and 3.8% of IBD patients had a negative antibody result compared to only 1.5% of controls (**Figure 1A**). After the second dose, the non-responder rate dropped to 17.1% in MM and 1.6% in SOT patients with different treatment regimens (**Table 3**), whereas all patients with IBD and controls showed a positive antibody result.

**Table 3:** Characteristics of non-responders at four weeks after the second dose.

Diagnosis n (%)	Ongoing therapy	Age mean ± SD (range)	Gender n (%)	Vaccine n (%)
SOT: (lung cancer) 1 (7.7)	chemotherapy	71 years	female	BNT162b2
MM:	either anti CD38, alkylating agents, thalodimide analogues, JAK inhibitors, cytostatic, proteasome inhibitors, steroids or a combination thereof; n=1 without ongoing treatment	73.4 years	female: 7 (58.3)	BNT162b2: 11 (91.7)
12 (92.3) (of those 4 (33.3) with stem cell transplantation)		± 6.5; (57 – 83)	male: 5 (41.7)	mRNA-1273: 1 (8.3)

Clinical and demographic parameters of those 13 patients with negative antibody responses measured at four weeks after the second vaccine dose.

Comparing antibody levels measured four weeks after the second dose, lowest geometric mean concentrations (GMC) were reached in MM patients (GMC=552.5 BAU/ml), as compared to IBD (GMC=2275.3 BAU/ml; p=0.0020) and controls (GMC=3205.5 BAU/ml; p<0.0001) (**Figure 1B**). In SOT patients (GMC=1529 BAU/ml), antibody levels were significantly lower than in controls (GMC=3205.5 BAU/ml; p=0.0107) with lower levels in the lung (GMC=694.9 BAU/ml) than in the breast cancer group (GMC=2096.4 BAU/ml; p=0.0061). With regard to age, only SOT patients below 60 years displayed higher S1-specific antibody levels (p=0.0052) than those 60 years and older (**Supplementary Figure 3A**). No significant differences in antibody levels between female and male participants were detected within the mentioned groups (**Supplementary Figure 3B**).

Subgroup analysis of IBD patients showed that four weeks after the second dose, patients treated with TNF-α inhibitors had significantly lower antibody levels (n= 59; GMC=1685 BAU/ml) than IBD patients without current immunosuppressive/-modulatory medication (n=21; GMC=3676 BAU/ml; p=0.0005), patients receiving the α4β7-integrin antagonist vedolizumab (n=19; GMC=3454 BAU/ml; p=0.0104) or controls (n=66; GMC=3206 BAU/ml; p<0.001) (**Figure 1C**). Lower antibody levels were found with all TNF-α inhibitors (infliximab: n=16, GMC=1319; adalimumab: n= 38, GMC=1799; golimumab: n=5; GMC=2246 BAU/ml) without significant differences between the three compounds.

Subanalysis according to the mRNA vaccine used revealed that in SOT as well as in MM patients, antibody levels were higher after vaccination with mRNA-1273 (SOT: GMC=2827 BAU/ml, MM: GMC=1289 BAU/ml) than with BNT162b2 (SOT: GMC=964.5 BAU/ml, MM: GMC=374.8 BAU/ml; p<0.05). With regard to non-responders, all (except one) were vaccinated with BNT162b2 (**Table 3**). For IBD and controls, only two and five individuals, respectively, were vaccinated with BNT162b2 not allowing for appropriate comparisons of outcome (**Figure 1D**).

We further evaluated neutralization titers in a subgroup 28.9% of participants (group for cellular analysis) and found a good correlation of S1-specific IgG measured by ELISA in all groups (p<0.01) (**Figure 1E**). Furthermore, none of the participants showed a positive neutralization titer before vaccination. After vaccination the geometric mean neutralizing titers (GMT) were significantly lower in SOT (n=8; GMT=99.9; p=0.0044) and MM patients (n=9; GMT=71.8; p=0.0073) compared to controls (n=25; GMT=453.3) (**Supplementary Figure 3C**). An additional analysis of ELISA-negatives in the neutralization test demonstrated that neutralizing antibodies were also not detectable (**Supplementary Figure 3D**).

### Cellular Immune Responses

**Figure 2:**
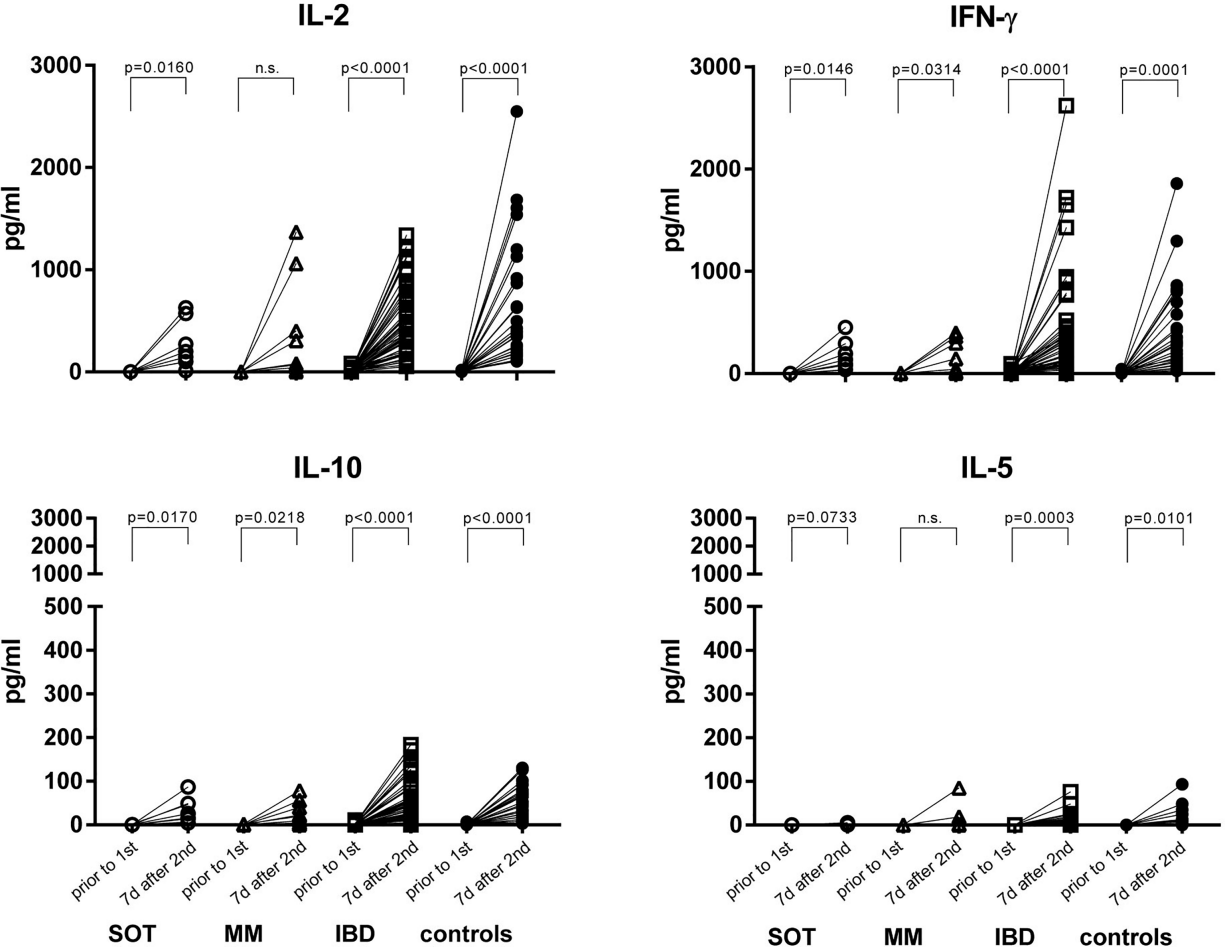
S1-spike-induced cytokine production before vaccination and one week after the second mRNA vaccine dose. PBMCs were stimulated with the peptide pool of the S1 subunit of the SARS-CoV-2 spike protein for 24 hours. Afterwards, the supernatants were collected and IL-2, IFN-γ, IL-10 and IL-5 were measured by the Luminex system. SOT (n=8) are represented as open circles, MM (n=9) as open triangles, IBD (n=52) as open squares and controls (n=26) as full circles. Differences between the groups below p values of 0.05 were regarded as significant. n.s., non significant.

In a preselected subgroup of participants, we analyzed the T cell response by using a cytokine release assay. After the second dose, IBD patients and controls, mounted a clear T cell response upon stimulation with the peptide pool of the S1 subunit. T cells of SOT patients secreted T cell growth factor IL-2, pro-inflammatory cytokines IFN-γ, IL-17a and GM-CSF and the regulatory cytokine IL-10, whereas only IFN-γ and concomitant IL-17a and IL-10 were induced in MM patients (**Figure 2** and **Supplementary Figure 4**).

**Figure 3:**
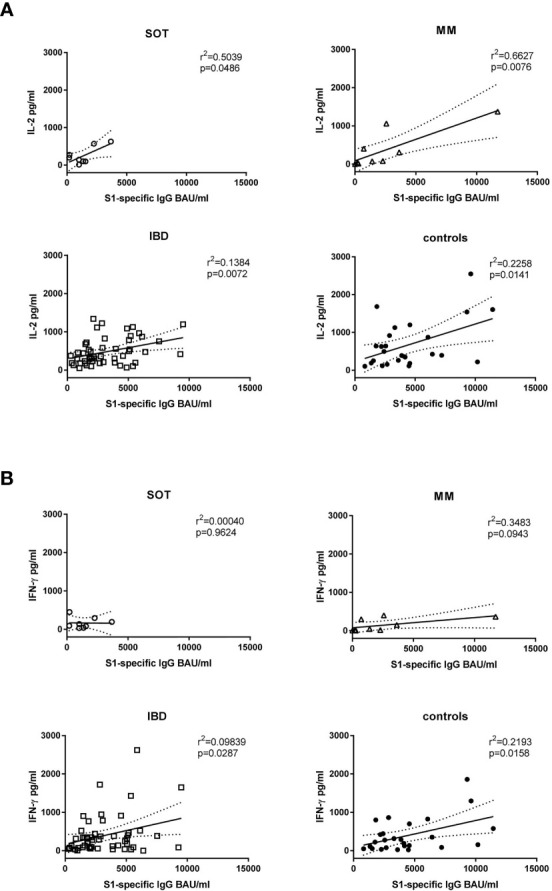
Correlation of antibody levels with cytokine production. The IL-2 **(A)** and IFN-γ **(B)** levels shown in **Figure 2** were correlated with the S1-specific IgG antibody levels of week four after the second mRNA dose. SOT (n=8) are represented as open circles, MM (n=9) as open triangles, IBD (n=52) as open squares and controls (n=26) as full circles. Differences between the groups below p values of 0.05 were regarded as significant. Dotted lines represent 95% confidence intervals.

Further, in controls and in IBD patients both IL-2 (r^2 ^= 0.1384, p=0.0072 and r^2^ = 0.2258, p=0.0141, respectively) and IFN-γ (r^2^ = 0.09839, p=0.0287 and r^2^ = 0.2193, p=0.0158, respectively) levels correlated positively with antibody levels. In SOT and MM patients this was true for IL-2 (r^2^ = 0.5039, p=0.0486 and r^2^ = 0.6627, p=0.0076, respectively) but not for IFN-γ concentrations (**Figure 3**; r^2^ = 0.00040, p=0.9624 and r^2^ = 0.3482, p=0.0943, respectively). Upon restimulation with peptide pools derived from the SARS-CoV-2 nucleocapsid antigen, cytokine levels did not increase after the second dose, confirming that the immune response was derived from vaccination rather than from natural infection (**Supplementary Figure 5**).

### Leukocyte Phenotypes

Phenotyping of leukocytes was performed in whole blood samples to determine the effects of the underlying diseases and the respective immunosuppressive/-modulating drugs on cellular compartments.

Patients with MM displayed lower total absolute cell numbers of leukocytes, total lymphocytes, CD3^+^ T cells and CD3^+^CD4^+^ T-helper cells compared to controls (p=0.0398, p=0.0601, p=0.0342 and p=0.0017, respectively) (**Supplementary Figure 6**). With regard to CD19^+^ B cells, patients with SOT (p=0.0381) and MM (p=0.0073) had lower levels than controls (**Supplementary Figure 6**). No significant differences were detected for monocytes, granulocytes, CD8^+^ T cells and NK cells, except for lower granulocyte counts in the MM patients compared to controls (p=0.0109) (**Supplementary Figure 6**). In contrast to cancer patients, we did not detect lower lymphocyte counts in IBD patients.

Correlation analysis showed that the B cell counts correlated with antibody levels at four weeks after the second dose in the total study population (**Supplementary Figure 7**; r^2^ = 0.03777, p=0.0014).

### Persistence of Humoral Immune Responses and Responses to Booster Vaccination

**Figure 4:**
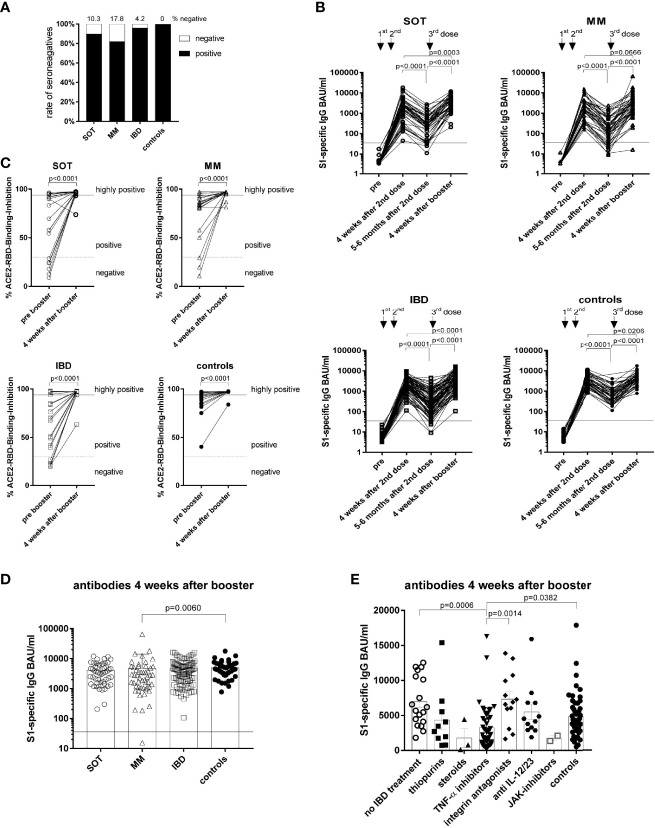
Antibody responses over a six months period and to booster vaccination. The percentage of seronegative participants at five-six months after the second dose **(A)**. Kinetics of antibody levels **(B)**, measured before the first, four weeks as well as five-six months after the second dose and four weeks after the booster dose for all study groups. Neutralizing antibody capacity before and after the booster dose in 20 randomly selected participants/group **(C)**. Differences in individual S1-specific IgG levels between the study groups **(D)**. S1-specific IgG levels of IBD patients in respect of their treatment and in comparison to the controls **(E)**. SOT (n=58) are represented as open circles, MM (n=56) as open triangles, IBD (n=114) patients as open squares and controls (n=62) as full circles. Differences between the groups below p values of 0.05 were regarded as significant. Black lines represent the threshold for positive results (35.2 BAU/ml) (A-B, D). Dotted lines represent cut-off for positive values and black lines the cut-off for highly positive values in the surrogate neutralization test **(C)**. Columns represent GMC with 95% confidence interval (CI)** (D)**.

Evaluation of antibody levels five-six months after the second dose demonstrated that the highest percentage of seronegatives belonged to the MM group (17.8%) followed by 10.3% in SOT and 4.2% in IBD patients with any kind of treatment, whereas all controls remained seropositive (**Figure 4A**, **Supplementary Figure 8A** and **Table 4**). Consequently, cancer and IBD patients had lower antibody levels compared to controls five-six months after the second dose (**Supplementary Figure 8B**)Furthermore, waning of antibodies (mean fold-decrease) was most impressive in MM (11.7; p=0.0024) and IBD patients (12.2; p<0.0001) followed by SOT patients (7.2; p=0.6505) compared to controls (5.4) (**Figure 4B**). With regard to IBD treatment, lowest antibody levels were detected in patients with TNF-α inhibitors (**Supplementary Figure 8C**). Furthermore, the fold-decrease of S1-specific antibodies was higher (p<0.001) in those with TNF-α inhibitors (19.0) than without TNF-α inhibitors (6.3).

**Table 4:** Characteristics of participants that became seronegative at five to six months after the second dose.

Diagnosis n (%)	Ongoing therapy	Age mean ± SD (range)	Gender n (%)	Vaccine n (%)
SOT: 4 (21.0) [lung cancer: 2 (10.5); breast cancer: 2 (10.5)]	antimetabolites, alkylating agents, anti-PD-1, mitotic inhibitors, hormones, steroids	70.3 ± 11; (52 - 81)	Female:4 (100), male:(0)	BNT162b2: 3 (75), mRNA-1273: 1 (25)
MM: 11 (57.9) (of those 6 with stem cell transplantation)	thalidomide analogue, proteasome inhibitor, steroids, anti-CD38; n=3 without ongoing treatment	66.5 ± 7.3; (57 - 80)	Female:3 (27.3), Male: 8 (72,7)	BNT162b2:7 (63.6), mRNA-1273: 4 (36.4)
IBD: 4 (21.1) [CD 3 (15.8); UC 1 (5.3)]	thiopurins, TNF-α inhibitors, steroids	47.5 ± 14; (31 - 68)	Female: 1 (25), male: 3 (75)	BNT162b2: 0, mRNA-1273: 4 (100)

Clinical and demographic parameters of those 19 patients with negative antibody responses measured at five-six months after the second vaccine dose.

The influence of the vaccine type on antibody levels was seen in cancer patients who maintained higher GMC´s even five-six months after primary vaccination with mRNA-1273 compared to BNT162b2 (SOT: 510.3 *vs*. 145.1, p=0.0001; MM: 214.6 *vs*. 139.8, p=0.0492) (**Supplementary Figure 8D**).

Importantly, booster vaccination five-six months after the second dose increased the antibody levels in all groups one month after the booster (p<0.0001; **Figures 4B-D**), whereby the antibody levels remained lower in the MM patients compared to the controls (p=0.0060) (**Figure 4D**). Accordingly, the neutralizing capacity of the antibodies increased (p<0.0001) in all groups after the booster (**Figure 4C**). Particularly, the GMCs reached after the second dose increased further in all groups after the booster dose (SOT: 1576 to 2982 BAU/ml; MM: 2248 to 3290 BAU/ml and controls: 3369 to 4145 BAU/ml) (**Figure 4B**). Notably, also all seronegative patients at five-six months after primary vaccination showing secondary vaccine failures i.e. lack to maintain antibody levels seroconverted again after the booster dose (**Figures 4B, C**).

## Discussion

In the present study, we aimed to explore humoral and cellular immune (non)-responsiveness following primary and booster vaccination with COVID-19-mRNA vaccines in a heterogeneous immunocompromised collective of patients with different immunosuppressive/-modulatory therapies.

The rate of antibody non-responders after the first dose was highest in patients with MM (50%) and SOT (28.6%), but improved four weeks after the second dose to 17.1% and 1.6%, respectively. These results underline the importance of completing the two-dose schedule with mRNA vaccines and are in line with data showing diminished seroresponses within one month after the second dose in MM and SOT patients compared to controls (18–20). Of note, patients with hematooncological cancers such as MM have seroconversion rates compared to SOT patients. In addition, also patients with IBD and controls showed a clear benefit from the second dose, as both reached 100% seroconversion albeit a low rate of non-responders after the first dose.

MM and SOT patients reached lower antibody levels than IBD patients and controls with lowest levels present in MM patients. Likewise, the most pronounced changes in lymphocyte counts were evident in MM patients with lymphopenia, demonstrated by diminished overall CD3^+^ T cells, CD4^+^ T helper cells and CD19^+^ B cells. These changes are typical for MM patients and their treatment (21) and might explain the lower seroresponses, since these cellular subsets are required for an effective immune response (22).

In both cancer patient groups (MM and SOT) stronger antibody responses were noted after mRNA-1273 vaccine, most likely due to the higher mRNA content in mRNA-1273 (100 µg of the full dose) as compared to BNT162b2 (30 µg). Likewise, higher antibody levels were reported for health care workers, referring to an immunocompetent cohort, and recently for IBD patients (23, 24). Further, a recent analysis showed that in a mainly immunocompetent cohort of vaccinees, mRNA-1273 is linked to lower risk of SARS-CoV-2 breakthrough infection and related hospitalization (25).Whether this observation may also indicate superior efficacy in these patients is also a limitation of this study, as clear cut-off values for protection are still not defined. Certain thresholds of antibody and neutralization titers have been published (26, 27), but thresholds may need to be adapted with emerging virus variants. However, determination of the quantity and quality of antibodies (WHO-benchmarked BAU values and neutralization titers) is already very useful to identify vaccine non-responders after the completion of primary vaccination and to immediately reinforce booster vaccination or further protection measures in these individuals.

When analyzing antibody responses in IBD patients in relation to their treatment regimens, only patients receiving TNF-α inhibitors exhibited lower antibody levels, but not those treated with integrin antagonist vedolizumab or the anti-IL-12/23 ustekinumab, which adds to a previous publication showing reduced GMTs in infliximab-treated patients compared to vedolizumab (28, 29). Results from the few study patients with JAK inhibitor upadacitinib, regarded as a potent immunosuppressive drug, may indicate a lower seroconversion rate, which is in line with a recent study showing up to 33% non-responders in upadacitinib-treated arthritis patients (30).

Vaccine-specific T cell reactivity to S1 measured by cytokine secretion was induced in all groups after two vaccine doses. However, in MM and SOT groups the levels were low in comparison to the IBD patients and controls. In general, IFN-γ and IL-2 levels correlated well with S1-specific antibody levels, indicating that high antibody levels are associated with clearly increased cytokine levels. However, a strong cellular response does not seem to be a prerequisite for the formation of antibodies, because some individuals with marginal cytokine induction did mount antibody responses well above the threshold for positivity. This dissociation between cellular and humoral responses was noticed particularly in SOT patients of our study. Differences in cellular and humoral responses were also reported in recent publications on vaccine responses after two doses of BNT162b2 in nursing home residents (31) as well as in rheumatoid arthritis patients treated with anti-CD20 antibodies who did not mount antibody production but significant IFN-γ levels (32). In this respect, IFN-γ production seems to be of importance, especially since IFN-γ has been linked with less severe courses in early COVID-19 infections (33). Therefore, analysis of T cell responses in antibody-non-responders may guide decisions regarding additional vaccine doses in immunocompromised individuals, however, by keeping in mind that an interpretation of T cell responses and their correlation with protection from COVID-19 is awaiting confirmation. As suggested also in a previous publication, those vaccine recipients lacking both antibody and cellular responses need to continue with non-pharmaceutical protection measures (34). In this regard, one limitation of our study was that in the preselected subgroup for the evaluation of T cell responses we only had one antibody non-responder included.

Concerning the persistence and waning of antibodies, particularly in MM patients, but also in SOT and IBD patients, antibody levels declined below the threshold of detection and lost neutralizing capacity within five-six months after the second dose. However, SOT and MM patients maintained their higher antibody levels when vaccinated with mRNA-1273 rather than with BNT162b2. Our data argue for booster vaccinations earlier than six months, not only for severely immunocompromised but also for patients on immunomodulatory drugs, as the third dose led to a strong increase of antibody levels and neutralizing capacity in all our patients´ groups, though antibody levels in the MM patients were lower than in controls. Importantly, also cancer and IBD patients that turned seronegative before six months after the second dose sufficiently responded to the booster dose, indicative for an established memory response. For IBD patients with TNF-α inhibitors we here show for the first time, that antibody levels are again lower even after application of the booster dose than in IBD patients without treatment and controls. In addition, we show that the booster dose increased peak antibody levels measured after the second dose in all study groups, which further argues for established B cell memory. In line with our data are other studies showing that memory B cells are stable for several months after primary vaccination and ready to respond after infection or booster vaccination and that booster doses can even broaden the neutralizing capacity also against other virus variants (35–37). Even though a reduced neutralizing activity of vaccine-induced antibodies against the Delta and Omicron variant has been observed in healthy individuals (38, 39), the vaccine-induced T cells responded equally well to the new emerging variants according to a recent data (40, 41) emphasizing the importance of timely boosters in patients with weakened vaccine responsiveness.

Taken together, our data show that non-responsiveness to primary SARS-CoV-2 mRNA vaccination mainly occurs in cancer patients, while IBD patients, who initially respond to the vaccine, show an early antibody loss particularly when treated with TNF-α inhibitors. Thus, our data argue for an mRNA booster dose already earlier than six months even in non-severely immunocompromised patients such as the IBD cohort. Additionally, our data also show that the vaccination with mRNA-1273 is more immunogenic in immunocompromised patients.

Furthermore, we further show a dissociation of humoral and cellular responses particularly in SOT patients, but that the existence of cellular responses is indicative for anamnestic vaccine responses following further vaccine doses.

Thus, we suggest immunomonitoring for the detection of non-responsiveness in immunocompromised patients along with the provision of individualized vaccination schedules for optimal medical care and disease prevention in these particularly vulnerable groups of patients.

## Data Availability Statement

The raw data supporting the conclusions of this article will be made available by the authors, without undue reservation.

## Ethics Statement

The studies involving human participants were reviewed and approved by Ethics Committee of the Medical University of Vienna (EK:1073/2021). The patients/participants provided their written informed consent to participate in this study.

## Author Contributions

Literature search: AWa, ST, CZ, GN, HS, WR, UW; Figures: AWa, ET; Study design: AWa, ST, WR, UW; Data collection: AWa, EG-S, A-MS, MO, BK, MaK, WFP, AO-R, LW, GT, LG, AWe, IZ ET, UW; Data analysis: AWa, A-MS, AO-R, LW, GT, LG, BK, WFP, EG-S, ET, MiK, HS, UW; Data interpretation: AWa, EG-S, AO-R, LW, GT, LG, WFP, MiK, ET, HS, GN, WR, CZ, UW; Writing: AWa, CZ, UW; Revising the manuscript: AWa, EG-S, A-MS, MO, AWe, IZ, AO-R, LW, GT, LG, ET, BK, MaK, ST, WFP, MiK, HS, GN, WR, CZ, UW. All authors contributed to the article and approved the submitted version.

## Funding

The study was sponsored and financed by the Medical University of Vienna – third party funding by the Institute of Specific Prophylaxis and Tropical Medicine. – AO-R and HS - acknowledge funding by the Austrian Science Fund (FWF, P 34253-B).

## Conflict of Interest

WP has received honoraria from Novartis, BMS and Roche; MiK: investigator initiated research grant from Pfizer, consulting fees from Valneva, Bluesky vaccines and Excientia outside the current study; GN has received consulting fees from AbbVie, MSD, Takeda, Gilead, Janssen, Sandoz, Pfizer, Astro Pharma, Falk Pharma, Ferring and Vifor; WR received fees from Abbvie, Algernon, Amgen, AM Pharma, AMT, AOP Orphan, Arena Pharmaceuticals, Astellas, Astra Zeneca, Avaxia, Roland Berger GmBH, Bioclinica, Biogen IDEC, Boehringer-Ingelheim, Bristol-Myers Squibb, Calyx, Cellerix, Chemocentryx, Celgene, Centocor, Celltrion, Covance, Danone Austria, DSM, Elan, Eli Lilly, Ernest & Young, Falk Pharma GmbH, Ferring, Galapagos, Gatehouse Bio Inc., Genentech, Gilead, Grünenthal, ICON, Index Pharma, Inova, Intrinsic Imaging, Janssen, Johnson & Johnson, Kyowa Hakko Kirin Pharma, Landos Biopharma, Lipid Therapeutics, LivaNova, Mallinckrodt, Medahead, MedImmune, Millenium, Mitsubishi Tanabe Pharma Corporation, MSD, Nash Pharmaceuticals, Nestle, Nippon Kayaku, Novartis, Ocera, OMass, Otsuka, Parexel, PDL, Periconsulting, Pharmacosmos, Philip Morris Institute, Pfizer, Procter & Gamble, Prometheus, Protagonist, Provention, Quell Therapeutics, Robarts Clinical Trial, Sandoz, Schering-Plough, Second Genome, Seres Therapeutics, Setpointmedical, Sigmoid, Sublimity, Takeda, Teva Pharma, Therakos, Theravance, Tigenix, UCB, Vifor, Zealand, Zyngenia, and 4SC; CZ has received consulting fees from Athenex, payments or honoraria from MSD, Imugene, AstraZeneca, Servier and Eli Lilly and has patents planed/issued or pending with Imugene; UW is PI of clinical studies sponsored by GSK, Novartis and Pfizer.

The remaining authors declare that the research was conducted in the absence of any commercial or financial relationships that could be construed as a potential conflict of interest.

## Publisher’s Note

All claims expressed in this article are solely those of the authors and do not necessarily represent those of their affiliated organizations, or those of the publisher, the editors and the reviewers. Any product that may be evaluated in this article, or claim that may be made by its manufacturer, is not guaranteed or endorsed by the publisher.
