# Supplementary material for: SARS-CoV-2-mRNA Booster Vaccination Reverses Non-Responsiveness and Early Antibody Waning in Immunocompromised Patients – A Phase Four Study Comparing Immune Responses in Patients With Solid Cancers, Multiple Myeloma and Inflammatory Bowel Disease
Source: Front Immunol. 2022 May 12;13:889138. doi: 10.3389/fimmu.2022.889138 (PMC9133631; doi:10.3389/fimmu.2022.889138)
Supplement: Supplementary file 1 [file Presentation_1.pptx]

## Slide 1
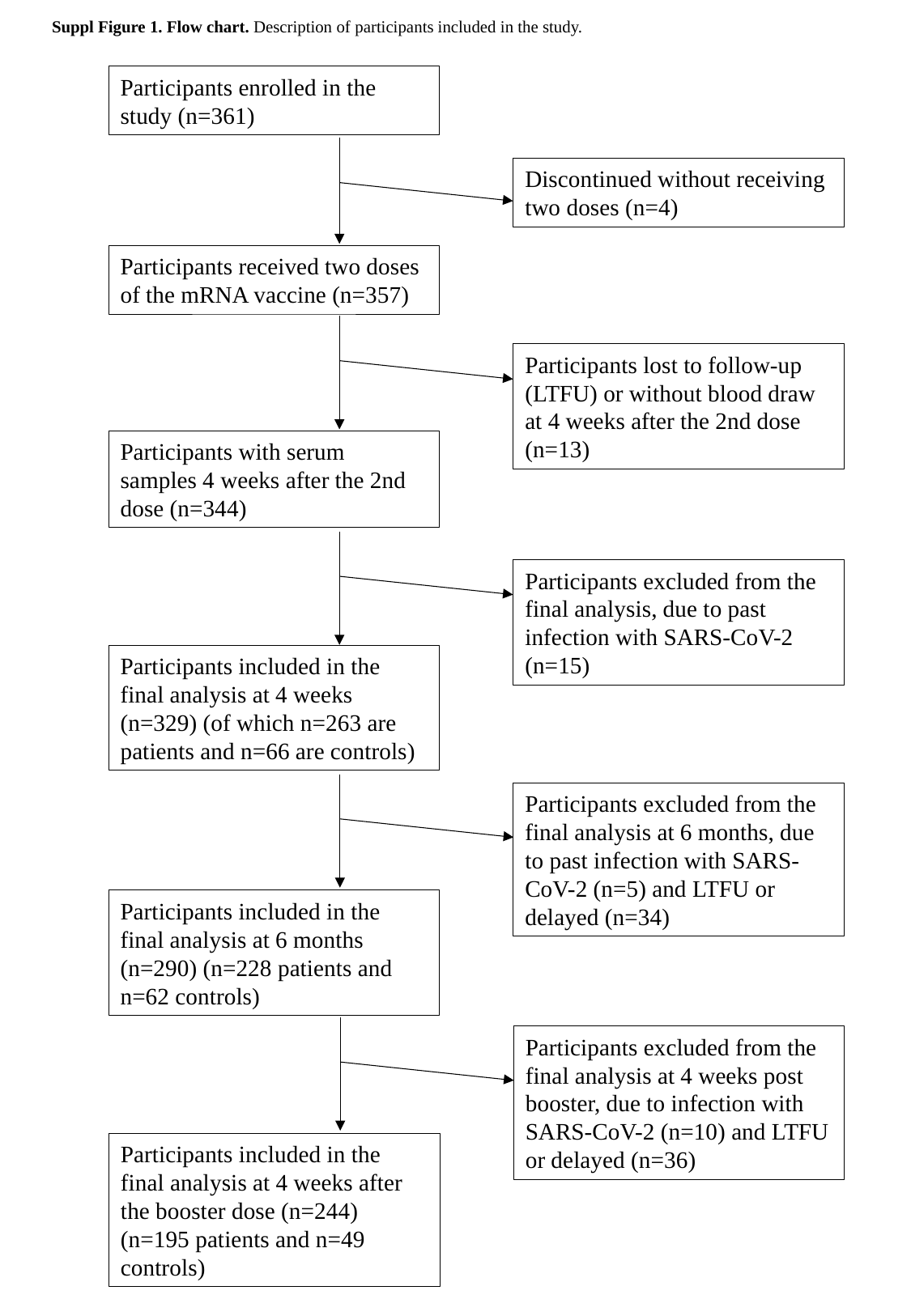

Suppl Figure 1. Flow chart. Description of participants included in the study.
Participants enrolled in the study (n=361)
Discontinued without receiving two doses (n=4)
Participants received two doses of the mRNA vaccine (n=357)
Participants lost to follow-up (LTFU) or without blood draw at 4 weeks after the 2nd dose (n=13)
Participants with serum samples 4 weeks after the 2nd dose (n=344)
Participants excluded from the final analysis, due to past infection with SARS-CoV-2 (n=15)
Participants included in the final analysis at 4 weeks (n=329) (of which n=263 are patients and n=66 are controls)
Participants excluded from the final analysis at 6 months, due to past infection with SARS-CoV-2 (n=5) and LTFU or delayed (n=34)
Participants included in the final analysis at 6 months (n=290) (n=228 patients and n=62 controls)
Participants excluded from the final analysis at 4 weeks post booster, due to infection with SARS-CoV-2 (n=10) and LTFU or delayed (n=36)
Participants included in the final analysis at 4 weeks after the booster dose (n=244) (n=195 patients and n=49 controls)

## Slide 2
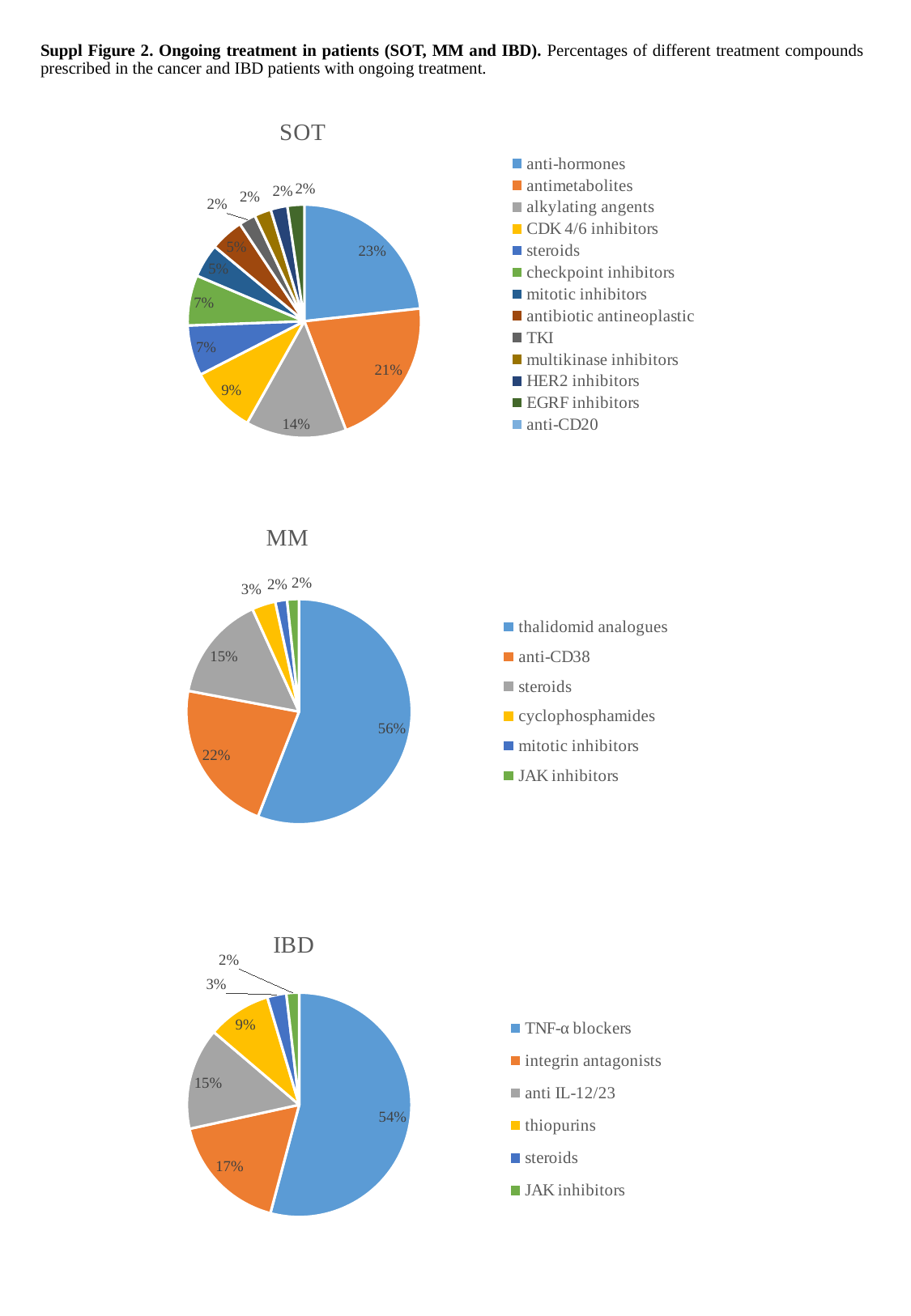

# Suppl Figure 2. Ongoing treatment in patients (SOT, MM and IBD). Percentages of different treatment compounds prescribed in the cancer and IBD patients with ongoing treatment.
### Chart: SOT
| Category | |
|---|---|
| anti-hormones | 10.0 |
| antimetabolites | 9.0 |
| alkylating angents | 6.0 |
| CDK 4/6 inhibitors | 4.0 |
| steroids | 3.0 |
| checkpoint inhibitors | 3.0 |
| mitotic inhibitors | 2.0 |
| antibiotic antineoplastic | 2.0 |
| TKI | 1.0 |
| multikinase inhibitors | 1.0 |
| HER2 inhibitors | 1.0 |
| EGRF inhibitors | 1.0 |
| anti-CD20 | 0.0 |
### Chart: MM
| Category | |
|---|---|
| thalidomid analogues | 33.0 |
| anti-CD38 | 13.0 |
| steroids | 9.0 |
| cyclophosphamides | 2.0 |
| mitotic inhibitors | 1.0 |
| JAK inhibitors | 1.0 |
### Chart: IBD
| Category | |
|---|---|
| TNF-α blockers | 59.0 |
| integrin antagonists | 19.0 |
| anti IL-12/23 | 16.0 |
| thiopurins | 10.0 |
| steroids | 3.0 |
| JAK inhibitors | 2.0 |

## Slide 3
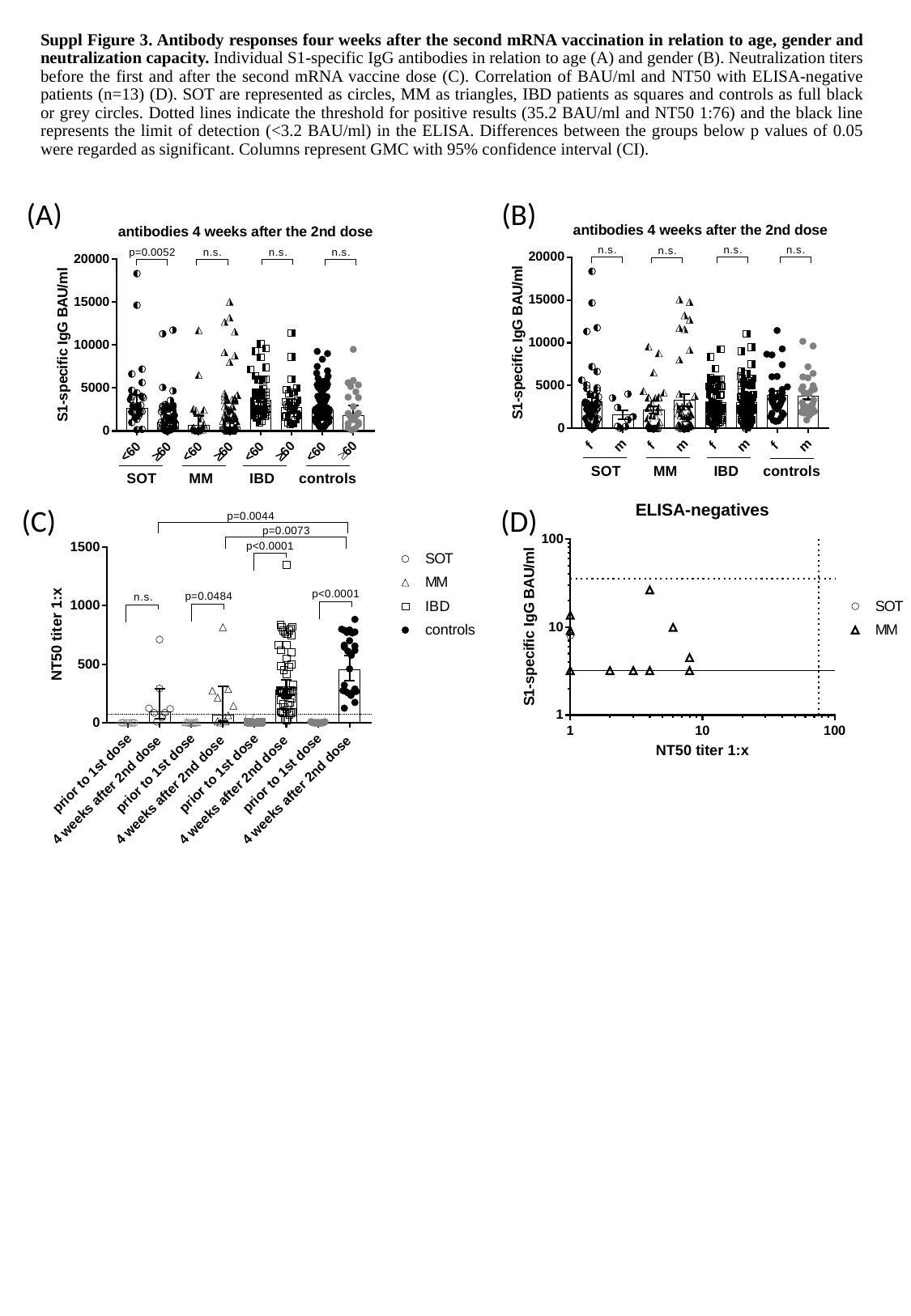

# Suppl Figure 3. Antibody responses four weeks after the second mRNA vaccination in relation to age, gender and neutralization capacity. Individual S1-specific IgG antibodies in relation to age (A) and gender (B). Neutralization titers before the first and after the second mRNA vaccine dose (C). Correlation of BAU/ml and NT50 with ELISA-negative patients (n=13) (D). SOT are represented as circles, MM as triangles, IBD patients as squares and controls as full black or grey circles. Dotted lines indicate the threshold for positive results (35.2 BAU/ml and NT50 1:76) and the black line represents the limit of detection (<3.2 BAU/ml) in the ELISA. Differences between the groups below p values of 0.05 were regarded as significant. Columns represent GMC with 95% confidence interval (CI).
(A) (B)
(C)
(D)

## Slide 4
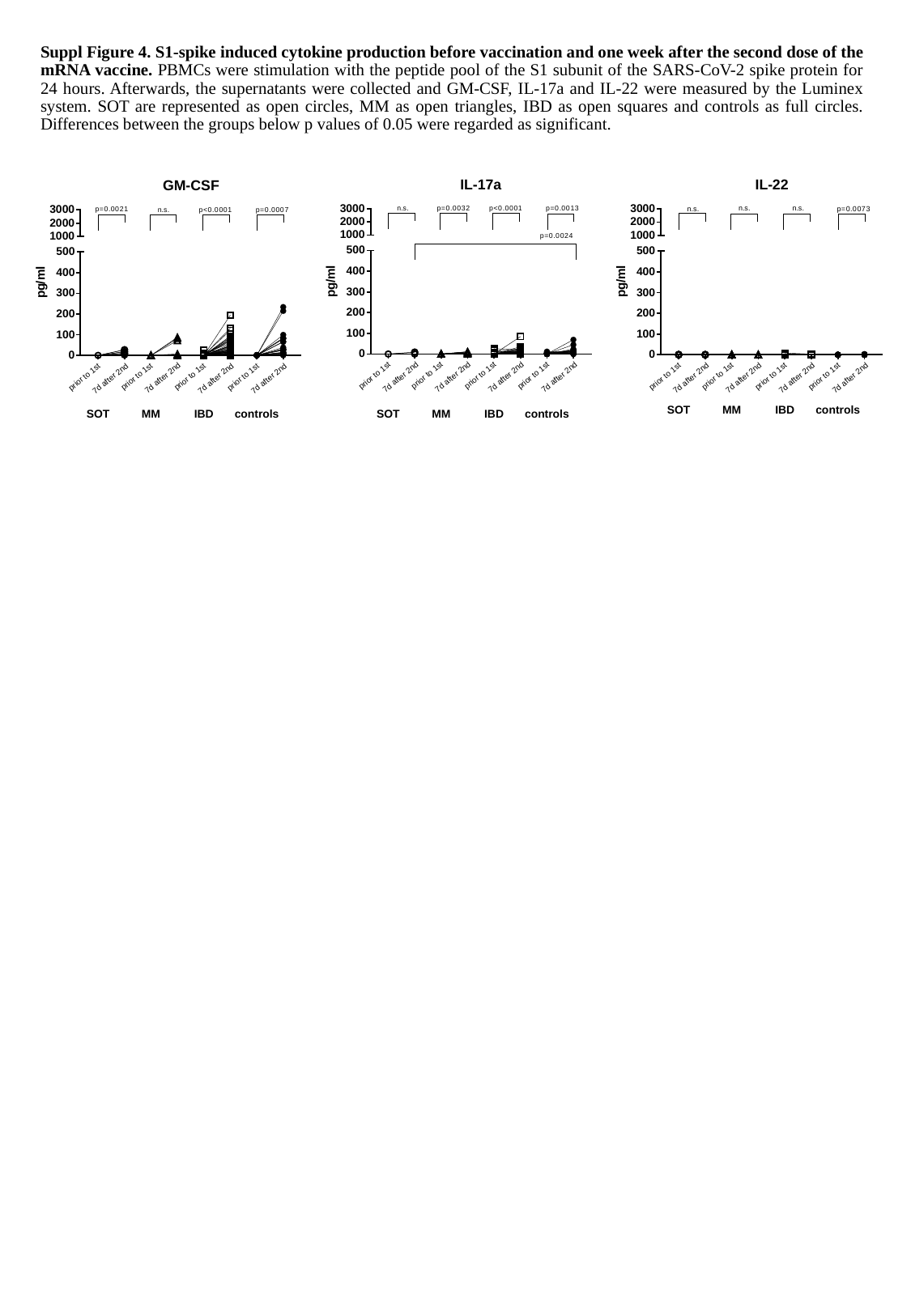

# Suppl Figure 4. S1-spike induced cytokine production before vaccination and one week after the second dose of the mRNA vaccine. PBMCs were stimulation with the peptide pool of the S1 subunit of the SARS-CoV-2 spike protein for 24 hours. Afterwards, the supernatants were collected and GM-CSF, IL-17a and IL-22 were measured by the Luminex system. SOT are represented as open circles, MM as open triangles, IBD as open squares and controls as full circles. Differences between the groups below p values of 0.05 were regarded as significant.

## Slide 5
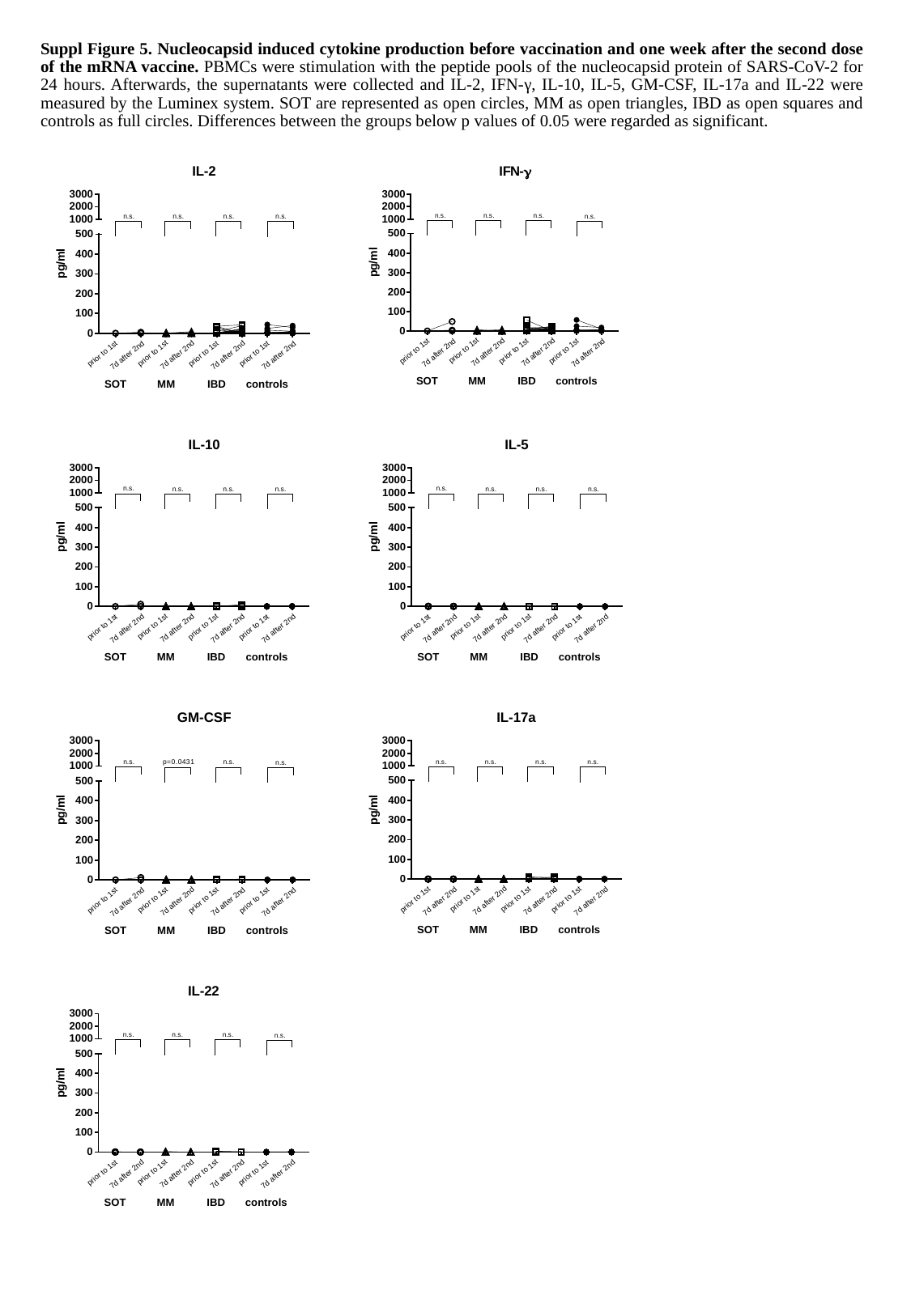

# Suppl Figure 5. Nucleocapsid induced cytokine production before vaccination and one week after the second dose of the mRNA vaccine. PBMCs were stimulation with the peptide pools of the nucleocapsid protein of SARS-CoV-2 for 24 hours. Afterwards, the supernatants were collected and IL-2, IFN-γ, IL-10, IL-5, GM-CSF, IL-17a and IL-22 were measured by the Luminex system. SOT are represented as open circles, MM as open triangles, IBD as open squares and controls as full circles. Differences between the groups below p values of 0.05 were regarded as significant.

## Slide 6
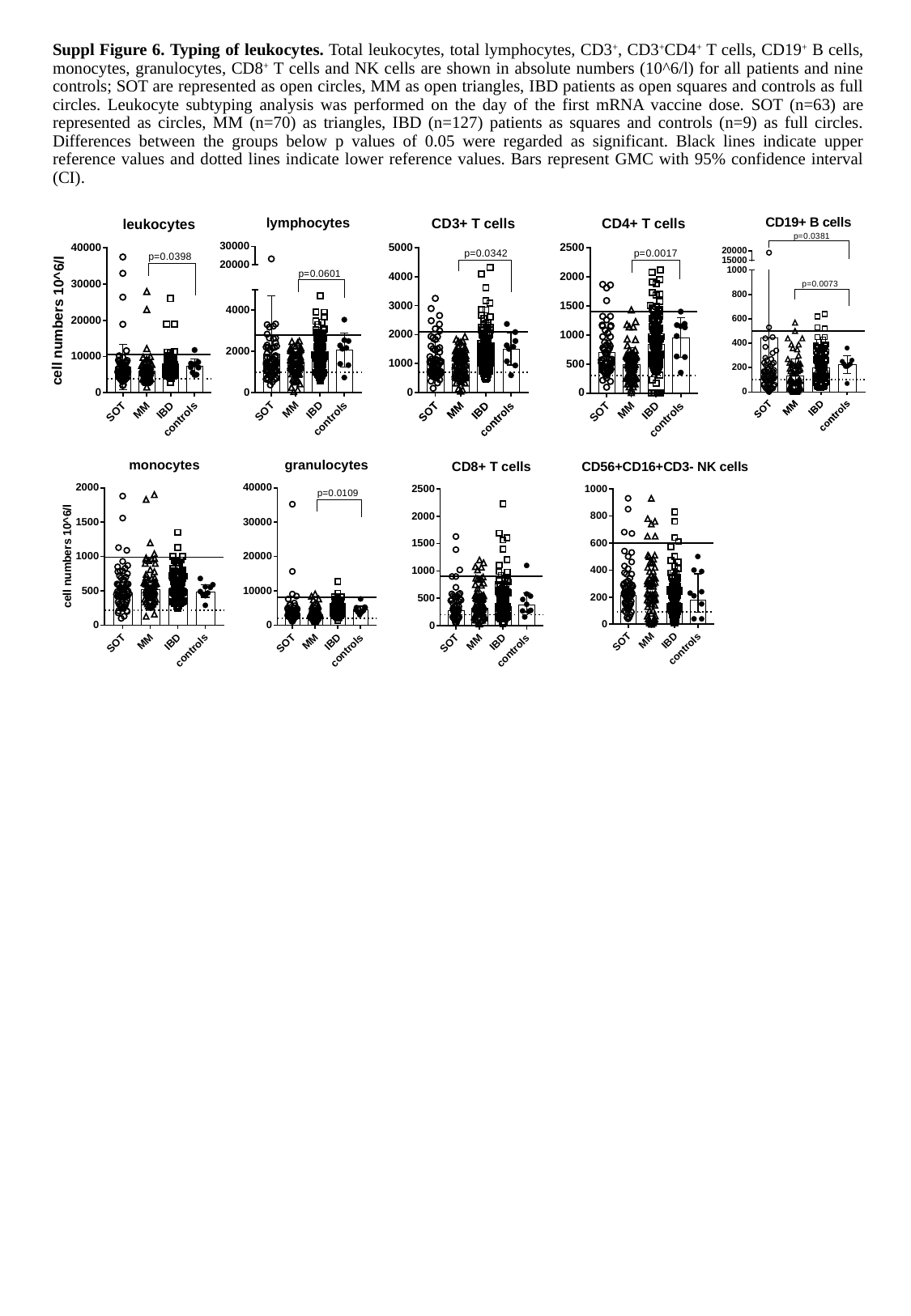

Suppl Figure 6. Typing of leukocytes. Total leukocytes, total lymphocytes, CD3+, CD3+CD4+ T cells, CD19+ B cells, monocytes, granulocytes, CD8+ T cells and NK cells are shown in absolute numbers (10^6/l) for all patients and nine controls; SOT are represented as open circles, MM as open triangles, IBD patients as open squares and controls as full circles. Leukocyte subtyping analysis was performed on the day of the first mRNA vaccine dose. SOT (n=63) are represented as circles, MM (n=70) as triangles, IBD (n=127) patients as squares and controls (n=9) as full circles. Differences between the groups below p values of 0.05 were regarded as significant. Black lines indicate upper reference values and dotted lines indicate lower reference values. Bars represent GMC with 95% confidence interval (CI).

## Slide 7
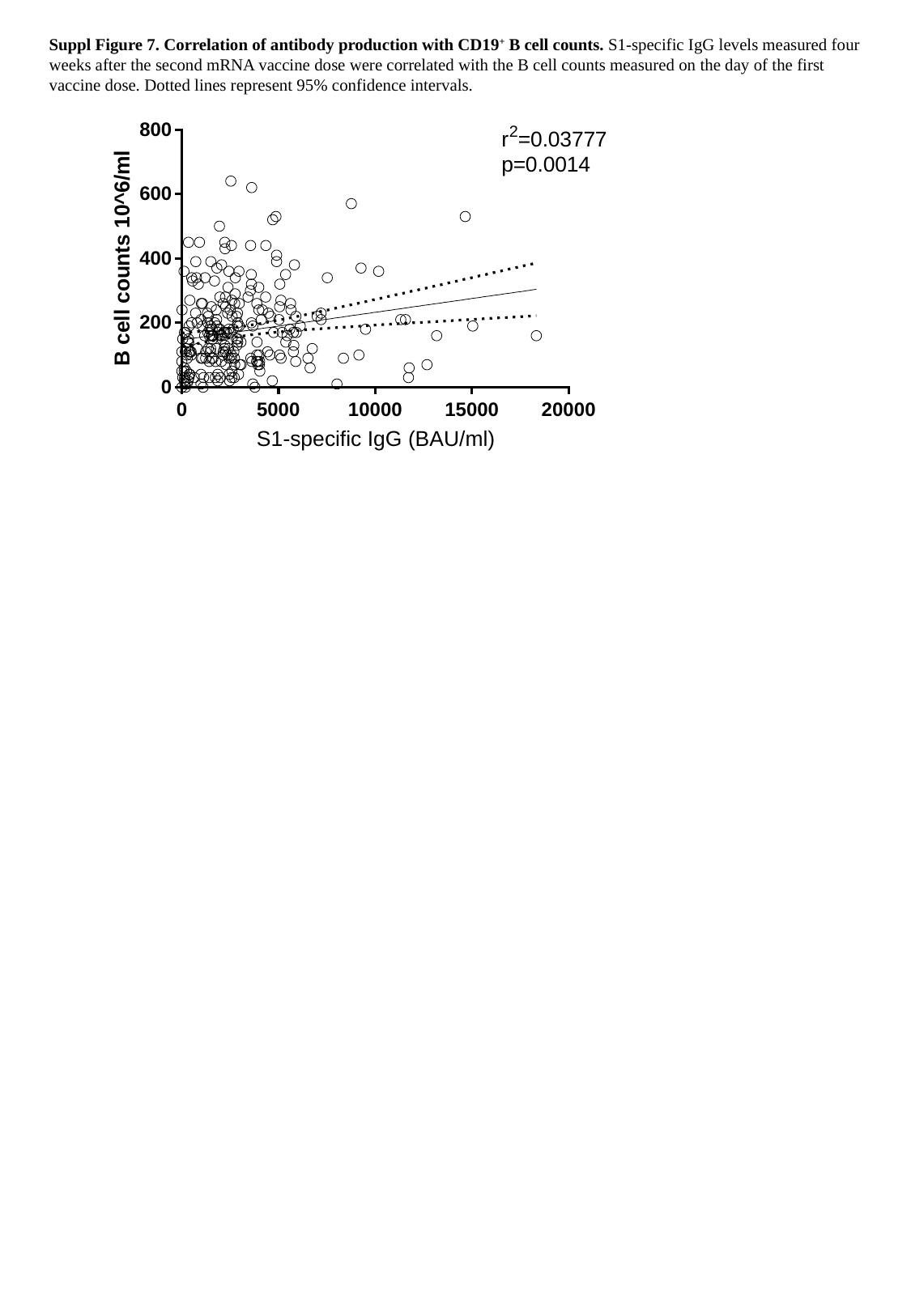

Suppl Figure 7. Correlation of antibody production with CD19+ B cell counts. S1-specific IgG levels measured four weeks after the second mRNA vaccine dose were correlated with the B cell counts measured on the day of the first vaccine dose. Dotted lines represent 95% confidence intervals.

## Slide 8
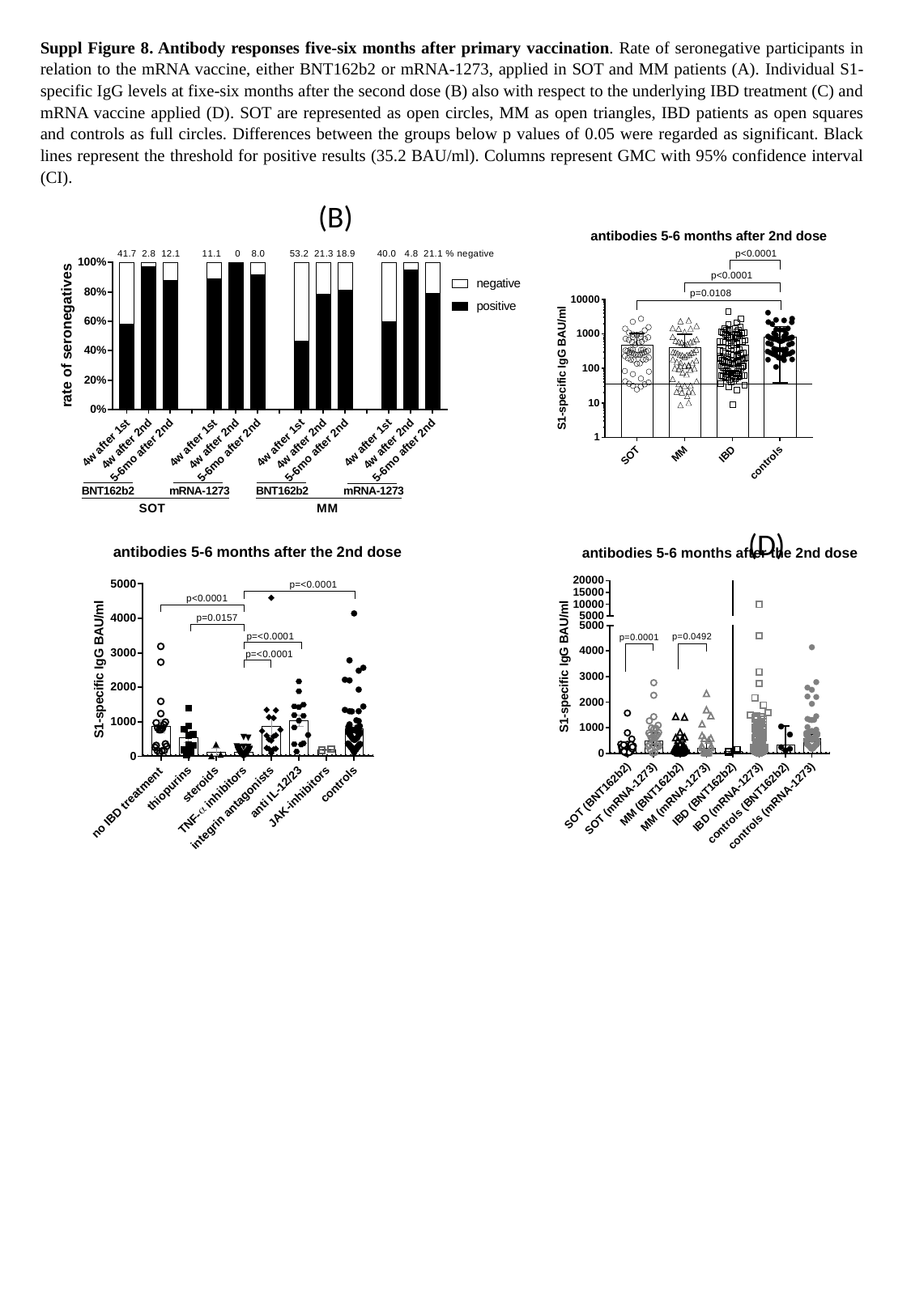

Suppl Figure 8. Antibody responses five-six months after primary vaccination. Rate of seronegative participants in relation to the mRNA vaccine, either BNT162b2 or mRNA-1273, applied in SOT and MM patients (A). Individual S1-specific IgG levels at fixe-six months after the second dose (B) also with respect to the underlying IBD treatment (C) and mRNA vaccine applied (D). SOT are represented as open circles, MM as open triangles, IBD patients as open squares and controls as full circles. Differences between the groups below p values of 0.05 were regarded as significant. Black lines represent the threshold for positive results (35.2 BAU/ml). Columns represent GMC with 95% confidence interval (CI).
(A) (B)
(C) 							 (D)
